# Effectiveness and safety of follitropin alfa (Ovaleap®) for ovarian stimulation using a GnRH antagonist protocol in real-world clinical practice: a multicenter, prospective, open, non-interventional assisted reproductive technology study

**DOI:** 10.1186/s12958-020-00610-2

**Published:** 2020-05-26

**Authors:** Peter Sydow, Norbert Gmeinwieser, Katrin Pribbernow, Christoph Keck, Inka Wiegratz

**Affiliations:** 1 VivaNeo Praxisklinik Sydow Berlin, Private Practice, Berlin, Germany; 2 Pharmalog Institut für klinische Forschung GmbH, Ismaning, Germany; 3 Kinderwunschzentrum Magdeburg, Private Practice, Magdeburg, Germany; 4 amedes MDL, Hamburg, Germany; 5 Kinderwunsch & Hormonzentrum Frankfurt – Am Palmengarten, Private Practice, Gräfstraße 97, 60487 Frankfurt, Germany

**Keywords:** Ovaleap, Biosimilar, Follitropin alfa, Recombinant human follicle-stimulating hormone, GnRH antagonist, User satisfaction

## Abstract

**Background:**

The use of recombinant human follicle-stimulating hormone (r-hFSH) in ovarian stimulation protocols for infertility treatment in assisted reproductive technology (ART) clinical practice is well established. More recent advancements include the availability of biosimilar r-hFSH products, which expand the choices available to healthcare practitioners and patients. Better understanding of how such a product contributes to routine clinical practice is valuable to help prescribers make informed treatment choices. The objective of this study was to examine the effectiveness and safety of ovarian stimulation (OS) with follitropin alfa (Ovaleap®) for routine IVF or intracytoplasmic sperm injection treatment in gonadotropin-releasing hormone (GnRH) antagonist cycles in real-world ART clinical practice.

**Methods:**

This non-interventional, multicenter, prospective study was initiated in 34 specialized reproductive medicine centers in Germany. Eligible women were 18–40 years old with a body mass index < 30 kg/m^2^, menstrual cycle 24–35 days and anti-Müllerian hormone ≥1 ng/mL, who were undergoing a first OS cycle exclusively with Ovaleap® during routine ART using a GnRH antagonist protocol. Primary effectiveness outcomes were number of retrieved oocytes after OS and clinical pregnancy rate (CPR). Secondary outcomes included fertilization rate, number of transferred embryos, live birth delivery rate, safety, and user satisfaction with the Ovaleap® pen.

**Result(s):**

Of 507 women screened, 463 received at least 1 dose of Ovaleap® and 439 had Visit 2 data (per protocol population; PPP). The mean(±SD) number of retrieved oocytes was 11.8 ± 7.2 (PPP). The CPR among women with documented embryo transfer was 41.3% (158/383), resulting in a live birth delivery rate of 31.6% (138/437) among PPP patients with available follow-up information. Overall, 8.6% (40/463) of women reported ≥1 adverse drug reaction. Ovarian hyperstimulation syndrome occurred in 23 (5.0%) patients, rated mild in 14 (3.0%), moderate in 8 (1.7%), and severe in 1 (0.2%). Patients reported high user satisfaction and high convenience with use of the Ovaleap® pen.

**Conclusion:**

The effectiveness and safety of OS with Ovaleap® in a GnRH antagonist protocol were extended to real-world ART clinical practice for the first time.

**Trial registration:**

Registered on 22 June 2016 (retrospectively registered) at ClinicalTrials.gov (NCT02809989).

## Background

Infertility treatment with assisted reproductive technology (ART) often includes ovarian stimulation (OS) with exogenous gonadotropins. Recombinant human follicle-stimulating hormone (r-hFSH) was developed as an alternative to urinary-derived FSH, allowing for greater availability, decreased variability, and possibly reduced risk of immunological reactions during infertility treatment [1–3]. Comparable effectiveness and safety of r-hFSH vs urinary gonadotropins have been demonstrated [4].

Ovaleap® (follitropin alfa, Theramex UK Ltd) is an r-hFSH developed as a biosimilar to Gonal-f®, administered subcutaneously by the use of a multidose, reusable, semi-automated pen injector and approved by the European Medicines Agency (EMA) in 2013 [5]. Ovaleap® is indicated for OS in women receiving ART, as well as for treatment of anovulation associated with polycystic ovary syndrome (PCOS) or hypogonadal hypogonadism and stimulation of spermatogenesis. To meet the requirements of a biosimilar [6, 7], similarity of Ovaleap® to Gonal-f® was demonstrated in comprehensive comparability studies of their physicochemical and biological characteristics, as well as efficacy, and safety [8–10]. These comparability studies demonstrate that a biosimilar r-hFSH and the corresponding reference biologic (Gonal-f®) share essentially the same active pharmaceutical ingredient (follitropin alfa) [9]. Ovaleap® demonstrated therapeutic equivalence to Gonal-f® for stimulating follicular development in a phase 3 multinational, multicenter, randomized, active-controlled, comparative study of infertile women using ART [11]. The study demonstrated that patients receiving Ovaleap® or Gonal-f® were equivalent in number of retrieved oocytes, the primary endpoint recommended by the EMA for evaluating clinical comparability [7], and showed comparable secondary efficacy and safety profiles following OS using a long GnRH agonist protocol. Safety and efficacy of Ovaleap® treatment were further demonstrated in an open-label, follow-up study of these patients with up to 2 additional Ovaleap® treatment cycles (in total 207 cycles) with a GnRH agonist [12].

Reports from clinicians within an IVF-Worldwide web-based survey indicated that while 67.3% reported awareness of biosimilar r-hFSH products, only 25.6% had experience with biosimilar products and 92% indicated they would like more information on these products [13]. One clinically relevant approach to providing more information is the examination of effectiveness and safety within real-world, routine clinical practice. The real-world effectiveness of Ovaleap® has not previously been evaluated, nor the effectiveness and safety of Ovaleap® treatment used with a GnRH antagonist protocol. GnRH antagonist vs long agonist protocols for ART have been examined for efficacy and safety outcomes with support for lower incidence of ovarian hyperstimulation syndrome (OHSS) with GnRH antagonist protocols [14–16].

The objective of this current multicenter, prospective, non-interventional study was to examine effectiveness of OS with Ovaleap® for routine in vitro fertilization (IVF) or intracytoplasmic sperm injection (ICSI) in real-world ART clinical practice using a GnRH antagonist protocol. The primary endpoints examined were number of retrieved oocytes and clinical pregnancy rate (CPR). Safety and user satisfaction with the Ovaleap® reusable semi-automated pen were also evaluated.

## Methods

### Study design and population

The study was a multicenter, prospective, open, non-interventional study that initially included 34 specialized reproductive medicine centers in Germany; the final number included in the present analysis was 33, due to one center not being able to participate. The study was conducted in accordance with Section 4 (paragraph 23, sentence 3) and Section 67 (paragraph 6) of the German Medicines Act (“Arzneimittelgesetz” – AMG) [17]. The protocol (XM17-WH-40103) was registered on ClinicalTrials.gov (ClinicalTrials.gov identifier: NCT02809989) and the publicly available register for non-interventional studies at the German Association of Research-Based Pharmaceutical Companies (Verband Forschender Arzneimittelhersteller e.V. [http://www.vfa.de/de/forschung/nisdb/]). It was reviewed by the appropriate ethics committees. Patients provided written informed consent prior to study participation and enrollment began March 2016 and ended May 2017.

To determine the number of patients to enroll, a sample size calculation was carried out based upon the estimated pregnancy rate from national data. It could be estimated that a study size of 400 women would be sufficient. This was carried out using PASS 11 software.

Study visits included information at screening and assessment at baseline (Visit 1), final maturation trigger, oocyte retrieval and embryo transfer (Visit 2), and the clinical pregnancy examination (Visit 3). Visit 2 involved multiple components, and patients who completed Visit 2 may not necessarily have had an embryo transfer. The observation period lasted approximately 6 to 8 weeks from the start of stimulation therapy. Patients with a sonographically verified intrauterine pregnancy (fetal heart and sac) were followed up until the end of the pregnancy or delivery of the baby/babies.

Patients eligible for inclusion were women undergoing IVF or ICSI following OS with Ovaleap® during routine ART and using a GnRH antagonist protocol. Duration of treatment was for one stimulation cycle. Inclusion criteria included patients age 18 to 40 years old, with body mass index (BMI) < 30 kg/m^2^, menstrual cycle duration 24 to 35 days, and anti-Müllerian hormone (AMH) ≥1 ng/mL (assessed within the previous 12 months), who were undergoing a first stimulation cycle for ART. Women with PCOS, endometriosis (American Fertility Society grades 3 and 4), uterine myoma (intramural > 4 cm, submucosal), or hydrosalpinx were excluded. Also excluded were women receiving combined application of IVF and ICSI or OS for fertility preservation.

### Treatment

The decision to treat a patient with Ovaleap® was made separately from the decision to include the patient in the study. As a first step and prior to patient assessment, the participating physicians were free to choose the stimulation drug and the protocol according to their experiences and preferences. If a physician chose to treat her/his patient with Ovaleap® in an antagonist protocol she/he then screened the patient and checked for inclusion/exclusion criteria as a second step. If the patient fulfilled the criteria and was willing to participate, she was finally eligible to be included in the study. FSH treatment, administered subcutaneously daily, typically began on the second or third day of the menstrual cycle, with the duration and dosage at the discretion of the physician. Dosage was adjusted according to ovarian response and continued until sufficient development of follicles (as indicated by serum estrogen and/or ultrasound examination and local practice). Suppression of an endogenous LH surge was achieved by a GnRH antagonist. Type of GnRH antagonist and type of oocyte maturation trigger were at the discretion of the treating physician. For final oocyte maturation and timing of oocyte retrieval, a single dose of recombinant human chorionic gonadotropin (r-hCG), urinary hCG was administered, or alternatively a GnRH agonist.

### Study assessments

The primary effectiveness endpoints included number of oocytes retrieved and CPR. Secondary effectiveness endpoints included total dose and administration duration of r-hFSH, serum estradiol level at the time of last examination prior to oocyte maturation trigger, endometrial thickness at the time of last sonography prior to trigger, drugs used for final oocyte maturation and timing oocyte retrieval, number of metaphase-II (MII) oocytes, percentage fertilization rate after IVF or ICSI, day of ovum pick-up (OPU), number of transferred embryos, and live birth delivery rate.

Safety and tolerability were examined by frequency and intensity (mild, moderate, severe) of adverse drug reactions (ADRs), defined as adverse events with an at least suspected relationship with Ovaleap®, including non-serious and serious ADRs. Frequency and intensity of OHSS were assessed at the discretion of the reporting physician.

Patient-reported outcomes for satisfaction with the Ovaleap® reusable semi-automated pen were evaluated with a previously described 7-question questionnaire [12, 18] after completion of FSH treatment (see Additional file 1; the document is available only in German language).

### Data analysis

The total treated population (TTP) included all patients who received at least 1 administration of Ovaleap®. The per protocol population (PPP) included all patients who received at least 1 administration of Ovaleap®, completed through to embryo transfer day (although did not necessarily have embryo transfer) and adhered to all study documentation criteria. Patients in whom all fertilized or unfertilized oocytes were frozen after OPU were not evaluated further. The planned analyses assessed outcomes using descriptive statistics (e.g., mean ± SD, median, range, percentages). A multivariate general linear model (GLM) analysis was performed to examine the effect of age (< 35 vs ≥35 years old), AMH level (≤2.5 ng/mL vs > 2.5 ng/mL), FSH total stimulation dose (≤1500 IU vs > 1500 IU), and FSH treatment duration (≤9 days vs > 9 days) on number of retrieved oocytes in PPP patients. The safety analysis included TTP patients. SAS version 9.4 was used for all statistical analyses.

## Results

Of the 507 women who were screened for enrollment in the study, 463 received at least 1 dose of FSH (TTP group) and 439 had Visit 2 data (PPP group) (Figure S1) (see Additional file 2). Among the PPP group, 56 women did not undergo an embryo transfer. Of these, the most frequent cause was a freeze-all intervention (24/56; 43%), followed by failed fertilization after either IVF/ICSI or degeneration of embryos (19/56; 34%), and no oocytes retrieved due to poor response or failed retrieval (13/56; 23%).

Demographic and baseline characteristics are shown in Table 1.

**Table 1:** Patient demographic and clinical characteristics

	TTP (***N*** = 463)	PPP (***N*** = 439)
Age, years, mean (SD)	32.2 (4.1)	32.1 (4.0)
BMI, kg/m^2^, mean (SD)	23.4 (3.6)	23.3 (3.2)
AMH, ng/mL, mean (SD)	3.6 (2.3)	3.6 (2.3)
Total r-hFSH dose, IU		
Mean (SD)	1651.2 (506.7)	1629.4 (479.9)
Median	1516.0	1500.0
Range	750.0–3825.0	750.0–3825.0
Duration of FSH stimulation, days		
Mean (SD)	9.5 (1.7)	9.5 (1.7)
Median	9.0	9.0
Range	4.0–17.0	5.0–16.0
GnRH antagonist protocol		
Missing, n (%)	1 (0.2)	0
No GnRH antagonist used, n (%)	8 (1.7)	0
Cetrorelix, n (%)	116 (25.1)	115 (26.2)
Days, mean (SD)	6.2 (1.9)	6.3 (1.9)
Ganirelix, n (%)	338 (73.0)	324 (73.8)
Days, mean (SD)	5.0 (1.7)	5.0 (1.7)
Serum estradiol prior to trigger, ng/L, mean (SD)	1362.0 (969.5)	1380.6 (968.8)
Endometrial thickness prior to trigger, mm, mean (SD)	9.7 (2.0)	9.8 (2.0)
Used for follicular maturation triggering		
Missing, n (%)	8 (1.7)	6 (1.4)
Recombinant hCG, n (%)	196 (42.3)	183 (41.7)
Urinary hCG, n (%)	198 (42.8)	190 (43.3)
GnRH agonist, n (%)	61 (13.2)	60 (13.7)

*AMH* anti-Müllerian hormone, *BMI* body mass index, *GnRH* gonadotropin-releasing hormone, *hCG* human chorionic gonadotropin, *PPP* per protocol population, *r-hFSH* recombinant human follicle-stimulating hormone, *SD* standard deviation, *TTP* total treated population

### Effectiveness outcomes

#### Number of oocytes retrieved

**Fig. 1:**
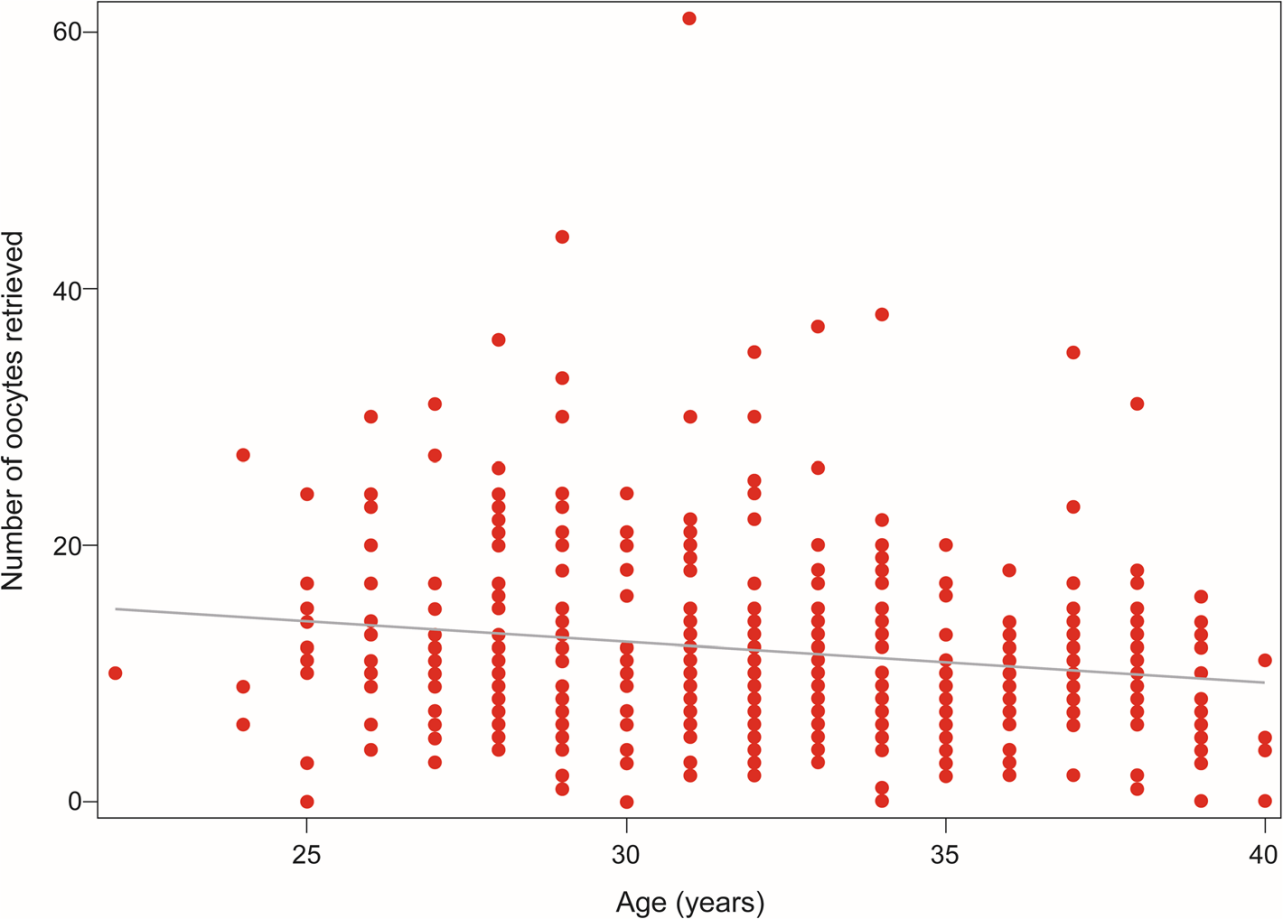
Number of retrieved oocytes by age in patients in the per protocol population

Mean numbers of retrieved oocytes were 11.8 in PPP patients, 12.1 in the subset of women undergoing ICSI, and 11.5 in the subset of women receiving IVF (Table 2). As expected, the number of oocytes retrieved decreased with age (Fig. 1). The multivariate GLM analysis found age ≥ 35 years and AMH ≤2.5 ng/mL were significantly associated with fewer oocytes retrieved (*P* = .0285 and *P* < .0001, respectively) in PPP patients.

**Table 2:** Primary and secondary effectiveness outcomes

	TTP (*N* = 463)^a^	PPP (*N* = 439)^a^
**Primary effectiveness endpoints**		
Number of oocytes retrieved		
Total, n	463	439
Mean (SD)	11.7 (7.2)	11.8 (7.2)
(median; range)	(10; 0–61)	(11.0; 0–61)
ICSI, n	331	314
Mean (SD)	12.1 (7.1)	12.1 (7.1)
(median; range)	(11.0; 1–61)	(11.0; 1–61)
IVF, n	117	115
Mean (SD)	11.4 (7.0)	11.5 (7.0)
(median; range)	(10; 2–38)	(10; 2–38)
No ART^b^, n	14	10
Mean (SD)	3.3 (7.0)	4.6 (8.0)
(median; range)	(0; 0–20)	(0; 0–20)
Missing, n	1	0
Mean (SD)	0 (NE)	–
(median; range)	0 (0–0)	–
CPR		
Pregnant, n (%)	165 (35.6)	158 (36.0)
Not pregnant, n (%)	298 (64.4)	281 (64.0)
CPR per embryo transfer, % (n/N)	41.4 (165/399)	41.3 (158/383)
**Secondary effectiveness endpoints**		
Number of 2PN oocytes		
Mean (SD)	5.8 (4.3)	5.9 (4.2)
(median;range)	(5.0; 0–33)	(5.0; 0–33)
Number of MII oocytes (ICSI patients only)		
N^c^	331	314
Mean (SD)	9.2 (5.2)	9.2 (5.2)
(median; range)	(8.0; 1.0–37.0)	(8.0; 1.0–37.0)
Percentage fertilization rate^d^		
Mean (SD)	67.8 (27.4)	68.3 (27.3)
(median; range)	(70.0; 0–300)	(70.0; 0–300)
Day of transfer		
Day 1, n/N (%)	1/399 (0.25)	0/383
Day 2, n/N (%)	74/399 (18.5)	72/383 (18.8)
Day 3, n/N (%)	150/399 (37.6)	147/383 (38.4)
Day 4, n/N (%)	43/399 (10.8)	43/383 (11.2)
Day 5, n/N (%)	124/399 (31.1)	115/383 (30.0)
Day 6, n/N (%)	7/399 (1.8)	6/383 (1.6)
Number of transferred embryos		
Mean (SD)	1.8 (0.4)	1.8 (0.4)
Median	2.0	2.0
Live birth delivery rate, n/N (%)^e^	143/460 (31.1)	138/437 (31.6)

*ART* assisted reproductive technology, *CPR* clinical pregnancy rate, *ICSI* intracytoplasmic sperm injection, *IVF* in vitro fertilization, *MII* metaphase-II (mature oocytes), *NE* non-estimable, *2PN* two pronuclei (normally fertilized oocytes), *PPP* per protocol population, *SD* standard deviation, *TTP* total treated population

^a^Unless otherwise indicated

^b^No ART carried out for any reason, information not given by study centers

^c^Performed in patients with ICSI

^d^Calculated as the percentage of 2PN oocytes out of the total number of MII oocytes

^e^Number of deliveries that resulted in a live birth among treated patients with follow-up information regarding live births

#### Clinical pregnancy

Overall, CPR per cycle was 35.6% (165/463) and 36.0% (158/439) in the TTP and PPP groups, respectively; CPR per embryo transfer was 41.4% (165/399) and 41.3% (158/383) in women with embryo transfer in the TTP and PPP groups, respectively (Table 2).

#### Secondary effectiveness outcomes

The mean number of MII oocytes was 9.2 (Table 2). Overall, fertilization rate was 68.3% (ICSI 69.6%; IVF 63.8%). The mean number of transferred embryos was 1.8. There were 138 live births in the PPP group who provided follow-up information (*n* = 437); thus, the live birth delivery rate was 31.6% (138/437) among patients with available follow-up information.

### Safety and tolerability

The overall frequency of ADRs was low (Table 3). OHSS was the most frequently reported non-serious ADR (16/463; 3.5%) and OHSS (7/463, 1.5%) and miscarriage (10/463, 2.2%) were the most frequently reported serious ADRs. There was 1 ectopic pregnancy.

**Table 3:** Patients with non-serious and serious ADRs in the total treated population (*n* = 463)

ADRs	n (%)
**Patient events**	
Patients with non-serious ADRs, total^a^	21 (4.5)
OHSS	16 (3.5)
Uterine polyp	2 (0.4)
Progesterone increased	1 (0.2)
Secondary hypothyroidism	1 (0.2)
Tachycardia	1 (0.2)
Blighted ovum	1 (0.2)
Patients with serious ADRs, total	19 (4.1)
Miscarriage	10 (2.2)
OHSS	7 (1.5)
Ectopic pregnancy	1 (0.2)
Hyperemesis gravidarum	1 (0.2)
**Babies born events**	
Patients with non-serious ADRs, total	0
Patients with serious ADRs, total	1 (0.2)
Hypoplastic left heart syndrome	1 (0.2)

*ADR* adverse drug reaction, *OHSS* ovarian hyperstimulation syndrome

^a^One patient experienced two non-serious ADRs (OHSS and uterine polyp)

Overall, OHSS was classified as mild (14/463, 3.0%), moderate (8/463, 1.7%), or severe (1/463, 0.2%). No cases of life-threatening OHSS were reported. The majority of patients (17/23; 73.9%), experiencing OHSS had AMH levels > 3.5 ng/mL.

### Patient-reported outcomes of pen use

Patients reported high levels of both user satisfaction and convenience with the Ovaleap® pen (full data not shown). In the PPP, 97.4% of patients reported they were sure or very sure about adjusting the daily dose of the drug, and 96.9% were sure or very sure that they had injected the correct dose. Instructional text was rated as easy or very easy to understand by 96.0% of women. Being satisfied or very satisfied with the Ovaleap® pen was reported by 99.8% of women, and the Ovaleap® pen was reported as convenient or very convenient to use by 99.0% of women.

## Discussion

This prospective, multicenter, non-interventional study extends the knowledge of the effectiveness of Ovaleap®, to a broader population of women, undergoing routine IVF/ICSI treatment, with a GnRH antagonist protocol. In the 2017 German IVF-Registry (DIR) annual report, 64.2% of patients received GnRH antagonist therapy, compared to 18.5% who received long GnRH agonist treatment overall [19]. Compared to long-term treatment with GnRH agonists, GnRH antagonist treatment is shorter, requires less FSH stimulation and fewer injections [14, 15], and is associated with a decreased risk of OHSS [16]. In poor responders and patients with PCOS, GnRH antagonist therapy has been proposed to be the first treatment protocol option [20]; however, a recent review article has also stated that a long GnRH agonist protocol is still applicable for poor responders [21].

The patient population studied here, based on mean age, is representative of the 30–34 year age range reported in 2018 DIR annual report, the most recent comprehensive analysis of trends in German IVF practice. This age range accounts for 30% of all oocyte retrievals done in Germany [22]. Amongst those patients aged 30–34 years in the 2018 report, the mean number of oocytes retrieved (IVF, 10.5; ICSI, 11.1) and CPR after embryo transfer (IVF, 40.0%; ICSI, 39.6%) are comparable to the findings presented here [22].

Additionally, in this non-interventional study of GnRH antagonist treatment, the primary endpoints of number of oocytes retrieved and CPR compared favorably with previous studies using a GnRH agonist protocol and in women of < 35 years old [11]. Other secondary endpoints associated with FSH drug usage were also similar [11]. OHSS was also comparable among the current non-interventional and the prior phase 3 comparative and open-label follow-up Ovaleap® studies [11]. Additionally, large multicenter studies of women with a similar age range as here, such as the Menopur in GnRH Antagonist Cycles with Single Embryo Transfer (MEGASET) study [23] using follitropin beta, reported 1.6% early-onset moderate/severe OHSS compared to here overall 1.9% moderate/severe OHSS with Ovaleap®, and in the recent Evidence-based Stimulation Trial with Human rFSH in Europe and Rest of World (ESTHER-1), utilizing follitropin delta and comparing to follitropin alfa in a GnRH antagonist protocol, the overall incidence of moderate/severe OHSS for follitropin alfa was 2.9% [24].

The primary endpoints in the current study are also comparable to previous randomized comparative r-hFSH studies examining follitropin alfa (Gonal-f®) [25–29], follitropin beta (Puregon®) [30] and more recent trials evaluating another biosimilar follitropin alfa [31] as well as follitropin delta [24]. In the analysis of the MEGASET study, follitropin beta was found to have comparable pregnancy and cumulative live birth rates when compared to menotropin (27% vs 30 and 38% vs 40%, respectively) [23]. Similarly, a study of 1050 Danish women found that treatment with GnRH antagonist or a GnRH agonist regimen resulted in comparable cumulative live birth rates, despite more oocytes being retrieved with the GnRH agonist protocol [32].

Comparison of the current non-interventional study’s number of retrieved oocytes (mean, 11.8) and CPRs among patients with embryo transfer (41.3%) with those of an observational, non-interventional study evaluating dosing regimens of follitropin alfa (Gonal-f®) in routine clinical practice demonstrated similar number of oocytes retrieved (11.4 ± 6.7) and CPRs (39.5%) [33]. Altogether, across study comparisons, the effectiveness of follitropin alfa (Ovaleap®) for OS during routine clinical practice using a GnRH antagonist protocol is supported.

Number of retrieved oocytes is a valuable primary endpoint as it has been associated with live birth rate [34, 35] and is not influenced by factors outside of r-hFSH stimulation in the manner that live birth rate may be influenced by other ART treatment factors (such as laboratory procedures, type of embryo transfer, regional policies of embryo transfer, differences in luteal phase support) [36]. The association with ongoing pregnancy and live birth rates is due to the availability of more oocytes rather than differences in oocyte quality [37, 38]. Notably, the live birth delivery rate achieved with Ovaleap® reported in our patient group of women with good prognostic factors (31.6% of the PPP among patients with available follow-up information) corresponds well to the birth rate in an “ideal” patient group (i.e. age < 36 years, at least 4 fertilized oocytes, fresh transfer on day 5, first or second IVF/ICSI cycle) receiving 2 embryos after IVF or ICSI in Germany from the 2016 DIR registry (32.0%) [19].

In view of the estimated global need for ART (≥1500 cycles/million population per year) [39], there is substantial need for infertility treatment options that are convenient, have high satisfaction and reduced burden, and possibly lower financial burden through expanded availability and healthcare options. In particular, the convenience, satisfaction, confidence in accurate dosing, and ease of use of the controlled OS device for treatment administration may play an important role in reducing ART treatment burden, which may improve patient treatment continuation [40–42]. Consistent with previous patient reports of the usability of the Ovaleap® pen [11, 12], almost all patients in the current study of Ovaleap® treatment during routine clinical practice rated the Ovaleap® pen with high satisfaction and high convenience, ease of use, and confidence in accuracy of dosing. These characteristics are consistent with expressed patient preferences during infertility treatment and may be helpful in reducing psychological stress and possibly improving treatment outcomes [43, 44].

Limitations of the current study are those associated with observational, non-interventional studies, including lack of randomization or controlled comparison, possible selection bias in the treated patients, and dependence on accurate reporting from the treating physicians. Strengths of the current study include the large patient sample, inclusion of a broader population of infertile women who may be more representative of the general population seeking ART treatment compared with the highly selected participants in randomized controlled trials, and greater representation of routine clinical practice in which treatment decisions are at the discretion of the treating physician. This approach has been advocated in order to provide clinical insights on newly introduced stimulation agents [45]. Altogether, the study findings of effectiveness, safety, and patient satisfaction with Ovaleap® treatment support Ovaleap® as a treatment option for women undergoing controlled OS for ART in routine clinical practice.

## Conclusions

Effectiveness, safety, and high user satisfaction with controlled OS with the Ovaleap® pen were extended to real-world ART clinical practice, including IVF and ICSI treatment, using a GnRH antagonist protocol. The real-world outcomes of this multicenter, prospective, open-label, non-interventional study are consistent with those previously reported in randomized controlled clinical trials.

## Supplementary information

**Additional file 1.** Patient satisfaction questionnaire.

**Additional file 2.** Patient disposition.

## Data Availability

The study was registered on 22 June 2016 (retrospectively registered) at ClinicalTrials.gov (NCT02809989), https://clinicaltrials.gov/ct2/show/NCT02809989. Study documents and individual participant data have not been shared.

## Abbreviations

ADR: Adverse drug reaction
AMH: Anti-Müllerian hormone
ART: Assisted reproductive technology
BMI: Body mass index
CPR: Clinical pregnancy rate
EMA: European Medicines Agency
GLM: General linear model
GnRH: Gonadotropin releasing hormone
ICSI: Intracytoplasmic sperm injection
IVF: in vitro fertilization
MII: Metaphase-II
OHSS: Ovarian hyperstimulation syndrome
OPU: Ovum pick-up
OS: Ovarian stimulation
PCOS: Polycystic ovary syndrome
PPP: Per protocol population
r-hCG: recombinant human chorionic gonadotropin
r-hFSH: Recombinant human follicle-stimulating hormone
TTP: Total treated population
