# Supplementary material for: Effectiveness and safety of follitropin alfa (Ovaleap®) for ovarian stimulation using a GnRH antagonist protocol in real-world clinical practice: a multicenter, prospective, open, non-interventional assisted reproductive technology study
Source: Reprod Biol Endocrinol. 2020 May 26;18:54. doi: 10.1186/s12958-020-00610-2 (PMC7251873; doi:10.1186/s12958-020-00610-2)
Supplement: Supplementary file 1 — Additional file 1. Patient satisfaction questionnaire. [file 12958_2020_610_MOESM1_ESM.pdf]

Fragebogen* zur Bewertung des Ovaleap®-Pens

Name der Patientin: ______________________________

Sehr geehrte Patientin,
bitte beantworten Sie alle nachfolgenden Fragen zum Ovaleap®-Pen und **kreuzen jeweils nur <u>eine</u> Antwortmöglichkeit an**. Der ausgefüllte Fragebogen verbleibt bei Ihrem Arzt / Ihrer Ärztin. Für Auswertung der Studie werden Ihre Antworten mit einer Patientennummer jedoch ohne Angabe Ihres Namens in elektronische Datenträger übertragen. Vielen Dank für Ihre Mitarbeit.

1. Wie sicher waren Sie sich, dass Sie die Tagesdosis der Medikaments genau eingestellt haben?

☐ **Sehr sicher** ☐ **Sicher** ☐ **Unsicher**

2. Wie sicher waren Sie sich, dass Sie die richtige Tagesdosis des Medikaments gespritzt haben?

☐ **Sehr sicher** ☐ **Sicher** ☐ **Unsicher**

3. Wie leicht waren die schriftlichen Anweisungen zur Anwendung des Pens zu verstehen?

☐ **Sehr leicht** ☐ **Leicht** ☐ **Nicht leicht**

4. Wie oft haben Sie den / die Mitarbeiter(in) im Studienzentrum darum gebeten, die Verabreichung der Tagedosis zu erklären?

☐ **Mehr als 4-mal** ☐ **4-mal** ☐ **3-mal**
☐ **2-mal** ☐ **1-mal** ☐ **Nie**

5. Wie würden Sie die allgemeine Benutzerfreundlichkeit des Pens auf einer Skala von 1 bis 10 bewerten, wobei "1" ganz leicht und "10" ganz schwierig bedeutet?

1	2	3	4	5	6	7	8	9	10
☐	☐	☐	☐	☐	☐	☐	☐	☐	☐

6. Wie praktisch war die Verabreichung der Studienmedikation mittels Pen für Sie?

☐ **Sehr praktisch** ☐ **Praktisch** ☐ **Unpraktisch**

7. Wie zufrieden waren Sie mit der Verabreichung der Studienmedikation mittels Pen?

☐ **Sehr zufrieden** ☐ **Zufrieden** ☐ **Unzufrieden**

*Fragebogen modifiziert nach Somkuti (2006):
Somkuti SG, Schertz JC, Moore M et al. Patient experience with follitropin alfa prefilled pen versus previously used injectable gonadotropins for ovulation induction in oligoanovulatory women. Current Medical Research and Opinion 2006; (10):198196.
