# Supplementary material for: Effectiveness and safety of follitropin alfa (Ovaleap®) for ovarian stimulation using a GnRH antagonist protocol in real-world clinical practice: a multicenter, prospective, open, non-interventional assisted reproductive technology study
Source: Reprod Biol Endocrinol. 2020 May 26;18:54. doi: 10.1186/s12958-020-00610-2 (PMC7251873; doi:10.1186/s12958-020-00610-2)

Effectiveness and safety of follitropin alfa for ovarian stimulation using a GnRH antagonist protocol in real-world clinical practice: a multicenter, prospective, open, non-interventional assisted reproductive technology study

Peter Sydow, Norbert Gmeinwieser, Katrin Pribbernow, Christoph Keck, Inka Wiegratz

---

Additional File 2. Patient disposition.

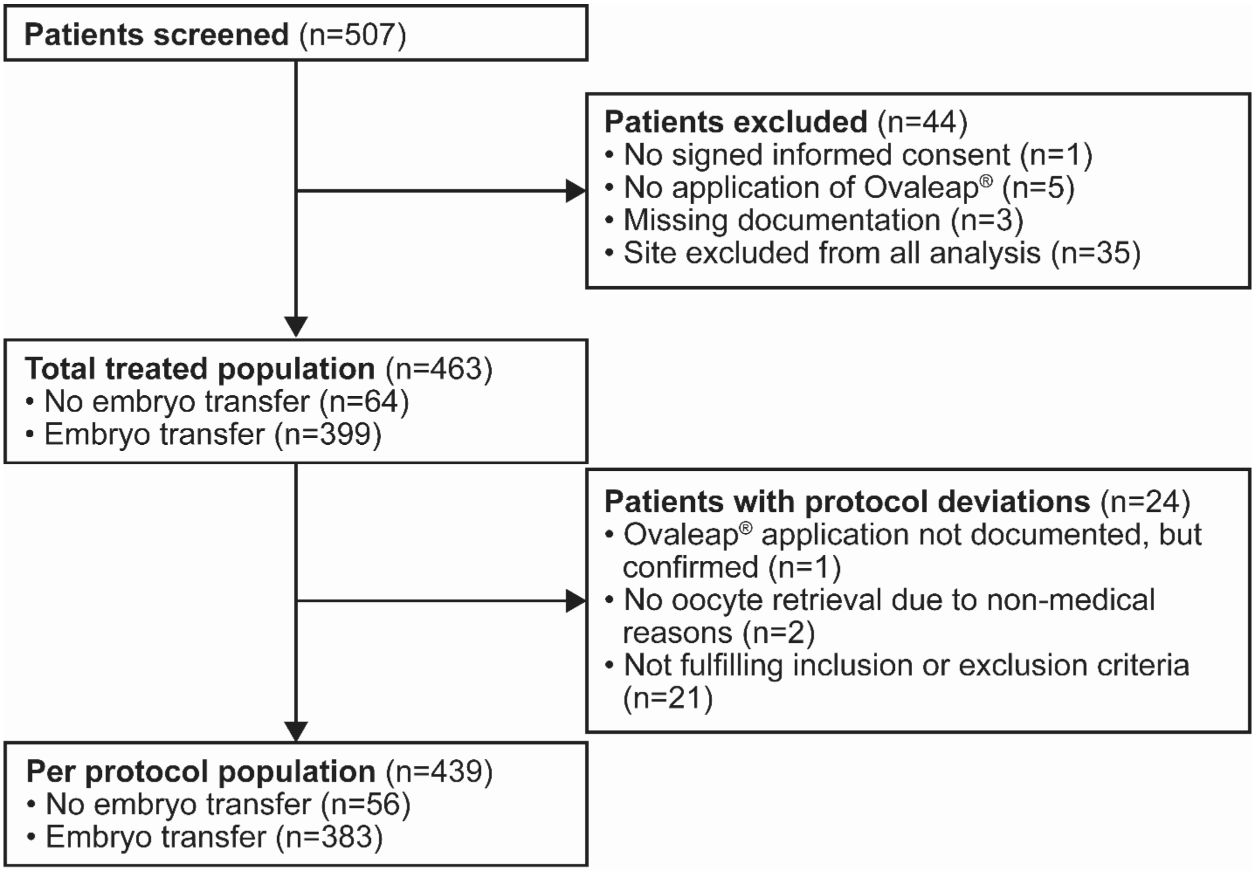

Supplement: Supplementary file 2 — Additional file 2. Patient disposition. [file 12958_2020_610_MOESM2_ESM.pdf]
